# Brachytherapy for primary nasal vestibule cancer using Au-198 grains

**DOI:** 10.1007/s13691-022-00546-x

**Published:** 2022-04-08

**Authors:** Hinako Harada, Yojiro Ishikawa, Shohei Tanaka, Keita Kishida, Rei Umezawa, Takaya Yamamoto, Noriyoshi Takahashi, Kazuya Takeda, Yu Suzuki, Keiichi Jingu

**Affiliations:** Department of Radiation Oncology, Tohoku University Graduate School of Medicine, 1-1 Seiryo-chou, Aoba-ku, Sendai, 980-8574 Japan

**Keywords:** Radiation therapy, Brachytherapy, Nasal vestibule cancer, Au-198

## Abstract

Radiation therapy (RT) is one of the definitive treatments for early-stage nasal vestibular carcinoma and has similar local control rates to resection surgery. There are various methods, including external beam RT and brachytherapy. This report describes a case who showed local control for more than 5 years after brachytherapy alone using Au-198 grains for nasal vestibular carcinoma. A 68-year-old Japanese man complained of swelling and pain in his left nasal cavity. He was diagnosed with squamous cell carcinoma (SCC) (cT1N0M0, stage I). An elevated mass of 8 mm in long diameter was found inside the left nasal cavity. Since the patient selected brachytherapy, nine Au-198 grains 185 mBq were permanently injected percutaneously under local anesthesia, and 85 Gy was prescribed. Grade three dermatitis was observed as an acute adverse event. After 2 years, mild telangiectasia of the left nasal skin and epilation of nasal hair in the left nasal cavity was regarded as late adverse events. The patient continues to keep a complete response for 5 years. For small nasal vestibular SCC, brachytherapy with Au-198 grains might be a good option.

## Background/introduction

Squamous cell carcinoma (SCC) of the nasal vestibule is a rare type of tumor that accounts for 1% of all the head and neck tumors and 3.8% of all nasal skin tumors [1, 2]. Radiation therapy (RT) is the recommended primary treatment for early-stage nasal vestibular carcinoma, and the local control rate is comparable to that of resection surgery [3]. External beam radiation therapy (EBRT), brachytherapy, or a combination of both are widely used with good functional and esthetic outcomes [2, 4–7].

There have been reports of low dose rate brachytherapy (LDRBT) using radium needles and EBRT combined with Cs-137; however, there have been no reports of nasal vestibular carcinoma treated by LDRBT using Au-198 alone [5, 7, 8].

In this report, we describe the case of nasal vestibular carcinoma treated by brachytherapy using Au-198 grains and achieved complete response for more than 5 years.

## Case presentation

A 68-year-old Japanese man complained of swelling and pain in his left nasal cavity 7 months before visiting our hospital. He had a history of hypertension and hyperlipidemia. He had a history of smoking (20 cigarettes per day for 46 years) and alcohol consumption (approximately 180 mL wisky per day for 46 years). His occupation was a construction worker, but his history of exposure to organic solvents was unknown. He visited a previous hospital, was diagnosed with nasal vestibulitis. The symptoms did not improve over the next 7 months despite prescribing a gentamicin ointment for observation, so he visited our hospital. As a result of a needle biopsy from a raised area in the left nasal cavity, a diagnosis of SCC was found.

There was an elevated mass about 8 mm in long diameter and 7 mm in height at 5 mm inside the left nasal cavity on gross examination. The right middle nasal concha showed redness and surface irregularity; however, skin invasion was not evident. There were no other apparent findings in the oral cavity or cervical lymph nodes. There were also no abnormal findings on nasopharyngeal fiber.

**Fig. 1:**
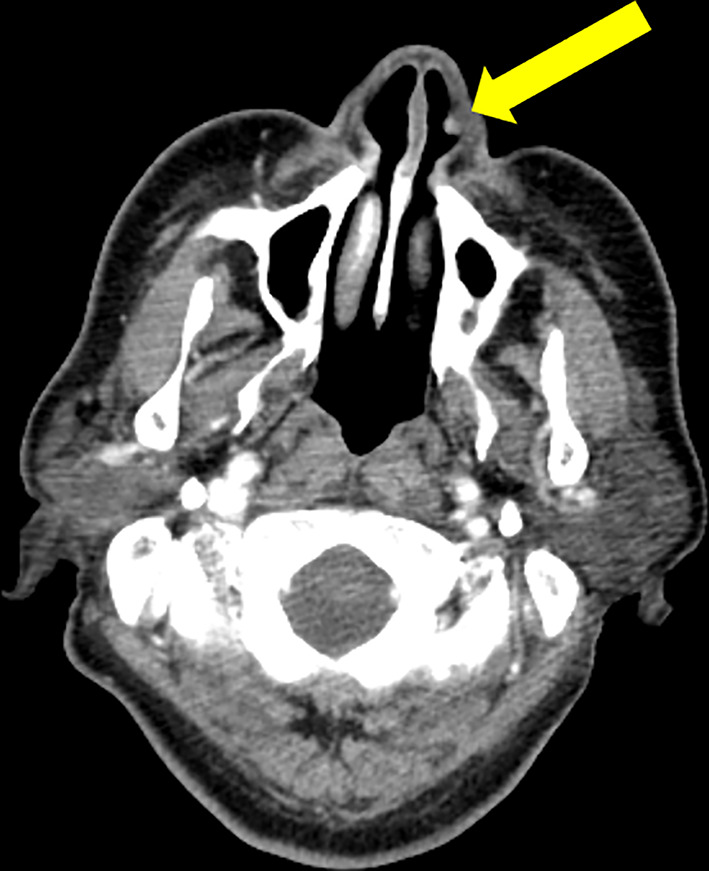
Axial enhanced computed tomography scan images of nasal vestibule show small elevated tumor (yellow arrow).

Computed tomography (CT) scan show small elevated tumor in the nasal cavity, and there were no enlarged lymph nodes or distant metastases (Fig. 1). Magnetic resonance imaging show same results as CT. There was only weak accumulation in the tumor area, and no distant metastasis was observed in positron emission tomography-computed tomography scan. The value of SCC antigen did not elevate in the blood test. Based on the above, the diagnosis of SCC of the nasal vestibule, cT1N0M0, stage I, was made (AJCC/TNM classification).

The patient was offered surgery, but he refused to receive surgery despite the fact that the patient had an Eastern Cooperative Oncology Group Performance Score of 0 due to concerns about the invasiveness and post-surgical cosmetic appearance. In addition, the patient did not request to undergo EBRT because of the long treatment period (about two months) and exposure to tissues other than the lesion. The patients selected LDRBT due to a low invasive procedure and short-term hospitalization, and we chose LDRBT using Au-198 grains because of the small size of the superficial lesion.

**Fig. 2:**
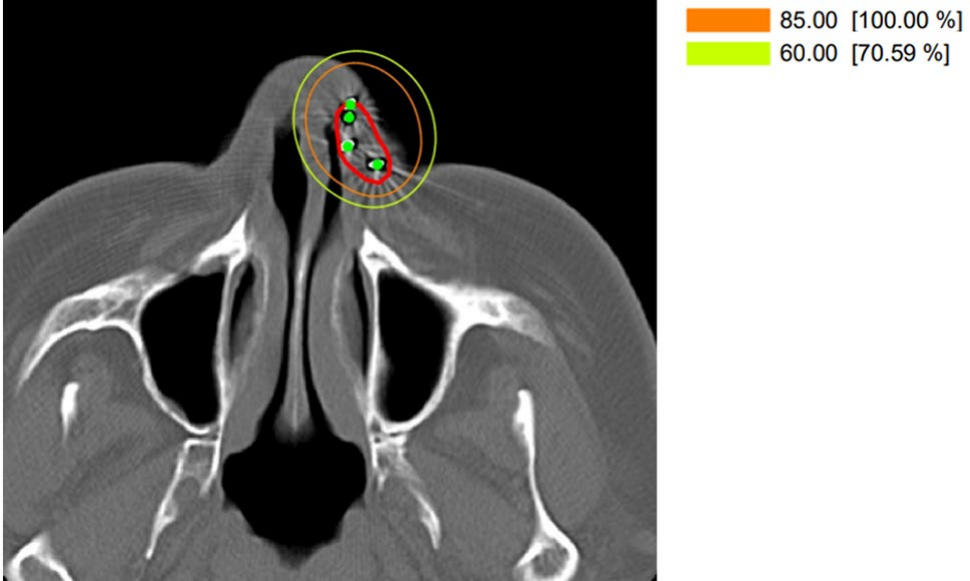
Dose distribution of brachytherapy for primary nasal vestibule cancer using Au-198 grains. Orange line shows 85 Gy (100% dose), yellow line shows 60 Gy (70.59%).

Under local anesthesia, we performed nine Au-198 grains 185 MBq were permanently punctured percutaneously. We set CTV to tumor + 1.0 cm, and the number of Au-198 grains was determined to be nine according to the volume. We made dose distribution by VariSeed (Varian) as a post-planning, and the calculated prescription dose using Modified Manchester system was 85 Gy covering the gross lesion with 5-mm margins (Fig. 2) [9]. For the dose, we referred to the recommendation by GEC-ESTRO/ACROP [10]. There were no complications due to the insertion, and the patient was discharged 5 days later. One month after that, redness, swelling and epidermolysis of the left nasal skin with a little pain were observed, and a diagnosis of Grade three dermatitis was made. The patient did not require any special treatment for the adverse events and improved spontaneously for a couple months.

**Fig. 3:**
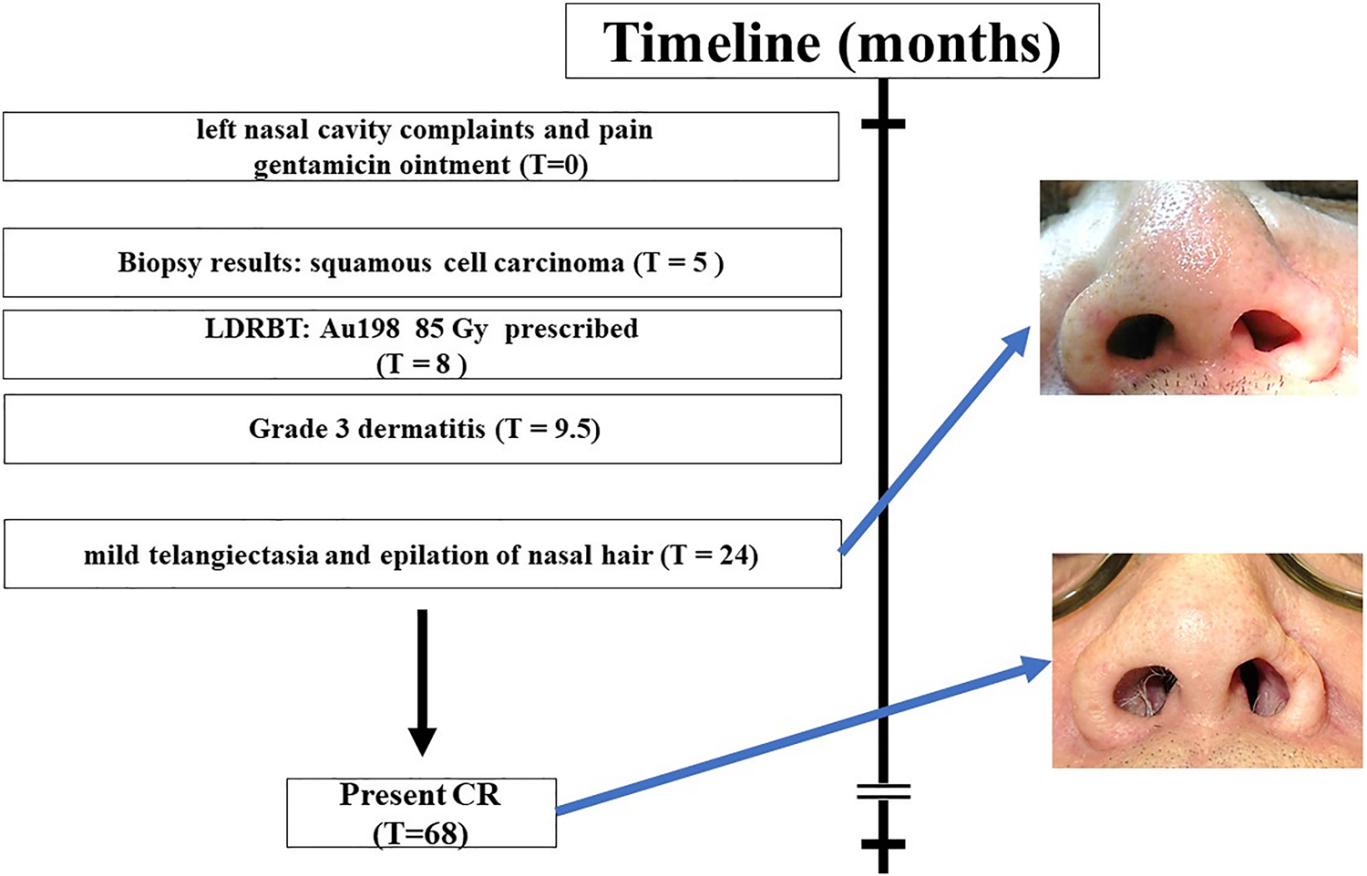
The timeline for intervention and clinical outcome is presented.

During the follow-up, mild telangiectasia of the left nasal skin and epilation of nasal hair in the left nasal cavity, which were late adverse events, were observed 1.5 years later. Five years later, the patient has kept complete response. The timeline for the present case is shown in Fig. 3.

## Discussion and conclusions

The nasal vestibule is defined as the part of the anterior nasal cavity that extends from the anterior opening of the nostrils to the curved ridge of the liminen nasi [3, 7].

It is believed that less than 5% of nasal vestibular cancers have lymph node metastasis at the time of initial diagnosis and that most cancers show localized disease in the nasal vestibule [3].

Surgery and/or RT are often the primary treatment for nasal vestibular carcinoma. However, there is no consensus on the optimal staging or approach. There are various options for RT, including EBRT, brachytherapy, or both, and there are many reports on the dose.

Masao K et al. reported a case of nasal vestibular carcinoma T1, 2 (Wang classification) treated with EBRT 40 Gy/16–20 fr followed by 18–20 Gy/4 fr/13–22 days HDRBT using Ir-192 [5]. In one of the four cases, the residual disease was found, and Au-198 grains were added, but in the other cases, no local recurrence was found in the long term.

Regarding BT and ERBT, Michal D. C. et al. reported the results of a retrospective comparison of outcomes after BT and EBRT for early-stage nasal cavity cancer. BT was superior to EBRT in terms of 3-year local control rate (95% vs. 71%, *p* < 0.01) and 3-year survival with preserved nose (SPN) (82% vs. 61%, *p* < 0.01). On the other hand, the incidence of CTCAE 5.0 grade two toxicity at 5 years was higher in the BT group (20% vs. 3%, *p* = 0.03), mostly due to radiation ulcers. Based on these results, the authors concluded that BT could be a first-line treatment for early-stage nasal cancer [11].

Michal D. C. et al. conducted a retrospective review of 102 patients with nasal vestibular carcinoma T1–T2 (Wang classification) at a single institution in a relatively large report [4]. Here, HDRBT was performed using Ir-192. It was concluded that the local control rate depended on the maximum tumor diameter. They reported that local control at 5 years was 100% when the tumor was less than 1.5 cm in length but decreased to 89% when the tumor was 1.5 cm or larger. Previously, Mak et al. also reported a longer recurrence-free period for tumors less than 1.5 cm in length [12].

There are various methods of brachytherapy, we chose Au-198 grains. Compared to other brachytherapy, Au-198 grains are more granular and smaller, making it easier to match the shape of the tumor. Other methods such as HDR allow only linear implantation, but Au-198 grains allow implantation according to the shape of the tumor. It may be suitable for small masses such as the one in this case.

In the present case, we chose LDRBT using Au-198 grains for a small mass of 8 mm in long diameter and thus long-term control and an excellent cosmesis could be achieved. To the best of our knowledge, there has been no report of treatment for SCC in primary nasal vestibule using Au-198 alone. We were able to achieve long-term local control with minimal late complication in this case, which can be attributed to the excellent dose-concentration properties of LDRBT.

## Conclusion

For nasal vestibular SCC, LDRBT with Au-198 grains might be an option for T1 lesions less than 1.5 cm in long length.

## Data Availability

The data include individual patient data, but the data are available from the corresponding authors upon reasonable request.
